# Nitrogen translocation by Highland cattle grazing in *Alnus viridis*-encroached pastures

**DOI:** 10.1007/s10705-023-10282-0

**Published:** 2023-04-10

**Authors:** Mia Svensk, Marco Pittarello, Pierre Mariotte, Ginevra Nota, Manuel K. Schneider, David Frund, Sébastien Dubois, Eric Allan, Massimiliano Probo

**Affiliations:** 1 Grazing Systems, Agroscope, Posieux, Switzerland; 2 Department of Ecology and Evolution, University of Bern, Bern, Switzerland; 3 Department of Veterinary Sciences (DSV), University of Turin, Turin, Italy; 4 Department of Agricultural, Forest and Food Sciences (DISAFA), University of Turin, Turin, Italy; 5 Forage Production and Grassland Systems, Agroscope, Zurich, Switzerland; 6 Feed Chemistry, Agroscope, Posieux, Switzerland

**Keywords:** Alps, Dung pats, GPS-tracking, Green alder, Livestock management, Nutrient translocation, Shrub encroachment

## Abstract

During the last decades, *Alnus viridis* has expanded over former montane pastures and meadows, due to land use and abandonment. This nitrogen-fixing woody species has triggered negative agro-environmental impacts, such as nitrogen (N) leaching, soil acidification and a reduced biodiversity. The aim of this study was to estimate the N translocation from *A. viridis*-encroached areas to adjacent open pastures by Highland cattle. In 2019 and 2020, Highland cattle herds equipped with GPS collars were placed in four *A. viridis*-encroached paddocks across Italy and Switzerland. The N content was measured in *A. viridis* leaves, herbaceous vegetation, and cattle dung pats, which were collected throughout the grazing season. Using GPS locations and collar activity sensors, livestock activity phases were discriminated. The N ingested by cattle was estimated through the N content of herbaceous vegetation and *A. viridis* leaves of vegetation patches visited by cattle during 24 h before dung sampling (N_24H_). The N content of herbaceous vegetation significantly increased with increasing *A. viridis* cover. The average N content in dung pats (31.2 ± 3.4 g.kg^−1^ DM) was higher than average values from literature on grazing cattle. Moreover, it was positively related to the N_24H_. Most of this N (29.5 ± 10.3 kg ha^−1^ yr^−1^) was translocated towards resting areas, which generally occurred on flat open pastures. Our results highlight the potential of Highland cattle to effectively translocate part of the ingested N from *A. viridis*-encroached towards targeted open areas, thus bringing new perspective for forage yield and quality improvement in the long-term.

**Supplementary Information:**

The online version contains supplementary material available at 10.1007/s10705-023-10282-0.

## Introduction

Encroachment of grasslands by woody species is a worldwide phenomenon (Eldridge et al. 2011; Wieczorkowski and Lehmann 2022). During the last decades, European mountain chains have faced major socio-economic transformations, above all on marginal areas, such as a decrease in agro-pastoral activities and land abandonment, with a consequent increase in shrub encroachment (MacDonald et al. 2000; Strebel and Bühler 2015; Orlandi et al. 2016). For instance, in Switzerland, shrub forests have increased by 22% between 1983 and 2017, with the greatest expansion recorded in the Alps (Abegg et al. 2020). In Central Europe, *Alnus viridis* (Chaix., D.C.) is the most rapidly expanding shrub species, as it is a pioneer species that has efficient sexual and vegetative reproduction traits (Farmer et al. 1985; Mallik et al. 1997; Caviezel et al. 2017). In Switzerland, its expansion rate is two to three times faster than the forest, and nowadays it represents 70% of the Swiss shrubland cover (Anthelme et al. 2007; Bühlmann et al. 2014; Abegg et al. 2020). This shrub can be found mostly in north and west facing steep slopes, but has recently started to expand in more gentle slopes (Caviezel et al. 2017). Its presence has led to many agro-ecological issues, since it can reduce some of the ecosystem services provided by montane grasslands. Indeed, its encroachment into open grasslands has led to a loss of potential agricultural production and has reduced landscape attractiveness, which has an important economic value for tourism (Ewald 2001). *A. viridis* encroachment also prevents forest succession by hindering montane conifers’ establishment (Hiltbrunner et al. 2014).

Because of its symbiosis with the N_2_-fixing actinomycete *Frankia alni* (Huss-Daniel 1997; Dawson 2008), the presence of *A. viridis* is leading to nitrogen enrichment in soils (Bühlmann et al. 2016). This creates a nitrogen saturated habitat which, combined with the reduction of light, temperature and the increased humidity under *A. viridis* canopy, facilitates the growth and dominance of a few shade-tolerant and nitrophilous species, such as *Adenostyles alliariae* (Gouan) A. Kern, *Cicerbita alpina L.* (Wallr.) and ferns (e.g. *Athyrium filix-femina* (L.) Roth and *Dryopteris dilatata* (Hoffm.), having low forage quality (Svensk et al. 2021). Therefore, encroached areas have lower plant and animal species richness if compared to adjacent open grasslands (Anthelme et al. 2001; Susan and Ziliotto 2004; Bühlmann et al. 2014; Cislaghi et al. 2019; Zehnder et al. 2020; Svensk et al. 2021).

Nitrogen fixation by *A. viridis* increases nitrification and thus enhances possibilities of nitrate and dissolved organic carbon leaching, leading to the pollution of streamlets and groundwater (Bühlmann et al. 2014, 2016; Hunziker et al. 2017).. Consequently, *A. viridis*-encroached habitats display higher risk of soil acidification, through the loss of base cations leached along with nitrates (Galloway et al. 2003; Bühlmann et al. 2016). Moreover, high nitrogen availability increases gaseous nitrogen loss such as the greenhouse gases NO and N_2_O (Galloway et al. 2003; Caviezel et al. 2014; Hiltbrunner et al. 2014).

Managing the expansion of *A. viridis* is thus an important goal for the restoration of the ecosystem services provided by montane pastures. Historically, until the 1950’s, the mechanical cutting of *A. viridis* for firewood combined with the higher grazing pressure at the time helped in controlling *A. viridis* spread (Caviezel et al. 2017). Nowadays, because of the loss of its economic benefit and the reduced workforce available in mountain areas, other more economically sustainable solutions have to be found to restore open pastures. Moreover, single cuts of *A. viridis* shrublands may lead to sprouting and thus denser stands, and may not be efficient to restore the below-ground conditions, as they are highly altered by the shrub encroachment (Schwob et al. 2017).

The use of targeted grazing to reduce tree and shrub-encroachment has already been recognized as being efficient and promising in the long-term (Mitlacher et al. 2002; Meisser et al. 2014; Elias et al. 2018). Because *A. viridis* leaves contain moderately high tannins levels (Stević et al. 2010), they can have low digestibility and palatability, preventing their use as a forage resource for production-oriented livestock (Kumar and Vaithiyanathan 1990; Besharati and Taghizadeh 2011). However, robust livestock breeds have the ability to digest lignified and tannin-rich vegetation through their tannin-tolerant rumen bacteria (Manousidis et al. 2016). Previous studies have shown that robust cattle breeds, such as Highland cattle, are able to feed on shrub species foliage with low forage quality (Pauler et al. 2020b; Svensk et al. 2021). The exploitation of woody species-encroached areas by Highland cattle can help controlling shrub and tree cover over time, by combining the effect of direct foliage consumption, trampling, and the mechanical breaking of branches, thanks to their long horns, and could potentially lead to the slow opening of the canopy, allowing the recolonization of typical pasture species in the long-term (Probo et al. 2016; Pauler et al. 2019, 2020a; Svensk et al. 2022).

In addition to their use to limit *A. viridis* encroachment, robust livestock could be used to balance the level of nitrogen in shrub-encroached areas and adjacent pastures. Indeed, nutrients can be transported through animal excretions, by taking in nutrients while foraging, and returning them through urine and dung excretions (Haynes and Williams 1993; Schnyder et al. 2010). Up to 95% of the nitrogen ingested by grazing animals can be excreted, mostly in urine (Whitehead 1970; Burggraaf and Snow 2010). Cattle activity (e.g., grazing, resting) is usually affected by topographic, vegetation and management factors (Probo et al. 2014; Homburger et al. 2015). Thus, the spatial distribution of dung pats and urine is not uniform and excretions are mainly deposited in resting areas, which are usually flat areas with low shrub cover (Costa et al. 1990; White et al. 2001; Kohler et al. 2006; Buttler et al. 2008; Koch et al. 2018). As a consequence, nutrients can be spatially redistributed from grazing areas, where they are taken in, to resting areas, where they are deposited (Kohler et al. 2006). Therefore, in *A. viridis* encroached pastures subjected to targeted grazing with Highland cattle, we expect an active N translocation from shrub-encroached to open and flat areas. *A. viridis-*encroached areas can have a high N level in the vegetation, due to the high N content of *A. viridis* leaves (Bühlmann et al. 2016) and that of the understory herbaceous vegetation. Indeed, the understory herbaceous vegetation is characterized by a significantly higher N content than that of both nutrient-rich and nutrient-poor pastures in the surroundings (Zehnder et al. 2017). Moreover, condensed tannins rich-species, such as *A. viridis,* may induce proportionally higher nitrogen excreted through dung than through urine (Burggraaf and Snow 2010), thus limiting ammonia volatilization from urea, and making nitrogen more available for plant utilization on the long-term (Lantinga et al. 1987; Berry et al. 2001).

The aim of this study was to evaluate whether Highland cattle grazing in *A. viridis*-encroached pastures can become a management tool to translocate N from shrublands to adjacent open pastures, and thus help reduce the negative environmental impacts of *A. viridis* expansion. Therefore, we studied Highland cattle herds grazing in Swiss and Italian *A. viridis*-encroached pastures and we measured the N content of the herbaceous vegetation, green alder leaves and cattle dung pats during two grazing seasons. Specifically, we aimed to: (i) measure the N content in Highland cattle dung pats and compare with literature data on grazing cattle dung pats; (ii) assess its relationship with the ingested N content during 24 h prior dung deposition; and (iii) estimate the N import–export fluxes within *A. viridis*-encroached areas and adjacent open pastures. We hypothesized that: (i) the N content in Highland cattle dung pats is higher than in dung pats from cattle grazing on open pastures and/or with similar crude protein-rich diets; (ii) the more often cattle have grazed in *A. viridis* encroached areas during 24 h before dunging, the more nitrogen-rich the dung pats are; and (iii) *A. viridis*-encroached areas have negative N fluxes (i.e., N is exported from these areas), while adjacent open pastures have positive N fluxes (i.e., N accumulates).

## Material and methods

### Study areas and grazing management

The study was conducted in 2019 and 2020 on four *A. viridis*-encroached paddocks grazed by two Highland cattle herds in the Swiss and Italian Alps (Table 1). The first paddock (paddock 1, 30.86 ha) was located in Val Vogna, Italy (province of Vercelli). The other three paddocks (paddock 2, 8.26 ha; paddock 3, 7.67 ha; paddock 4, 7.04 ha) were located in Bovonne, Switzerland (canton of Vaud) and were grazed in rotation by the same herd. All paddocks were grazed at a comparable stocking rate and had similar topographical conditions (Table 1). The four paddocks were representative of an *A. viridis* cover gradient, with an average cover of 26%, 51%, 61% and 71% respectively in paddock 1, 4, 2 and 3. Paddock 1, 2 and 3 were grazed during two summer seasons (2019 and 2020), while paddock 4 was grazed during one summer season (2020). More detailed information on the vegetation characteristics of paddocks 1, 2 and 3 can be found in Svensk et al. (2021). Each year, the herds grazed from the middle of June to the beginning of September. All the herds included cow/calf pairs and heifers, varying in age from 6 months to 17 years (with an average of five years for paddock 1, and four years for paddock 2, 3 and 4) and about 70% of the animals were present in both years at the same site. A water trough was installed in paddocks 2, 3 and 4, while natural streams were present in paddock 1 for the entire grazing period. As described in Svensk et al. (2022), in the second year of the project (2020), five molasses-based blocks were installed in small highly encroached areas of paddocks 1, 2, and 3, as part of another experiment set, to attract Highland herds into these areas. In each herd and during both years, six to ten cows were equipped with GPS collars (Followit AB ©, Tellus GPS System collars, Sweden) that recorded their position every ten minutes during the whole grazing period, with an accuracy of two to five meters. The GPS collars also recorded cattle neck movements through activity sensors of the X and Y axes.

**Table 1:** Topographical and management characteristics of the four *A. viridis*-encroached paddocks

Coordinates	Paddock 1	Paddock 2	Paddock 3	Paddock 4
	N45° 46′ 18.8" E7° 54′ 9.1"	N46° 16′ 9.8" E7° 6′ 44.2"	N46° 16′ 12.1" E7° 6′ 58.8"	N46° 16′ 15.9" E7° 07′ 02.8"
Elevation (m a.s.l.)	1897 ± 67	1745 ± 46	1789 ± 32	1877 ± 21
Slope (°)	21	23	21	23
Cover of A. *viridis* (%)	26	61	71	51
Grazable area (ha)	30.9	8.26	7.67	7.04
Number of vegetation patches	66	12	13	24
Size of vegetation patches (ha)	0.47 ± 0.06	0.69 ± 0.1	0.59 ± 0.08	0.29 ± 0.09
Number of grazing days 2019	44	17	18	–
Number of grazing days 2020	29	17	18	19
Livestock units^a^ 2019	45.4	29.8	29.8	–
Livestock units^a^ 2020	70.4	29.6	29.6	25.4
Stocking rate^b^ 2019	0.177	0.168	0.192	–
Stocking rate^b^ 2020	0.181	0.167	0.190	0.187
Number of GPS collars 2019	6	8	8	8
Number of GPS collars 2020	8	10	10	10

^a^LU, Livestock Unit. One livestock unit = 1 animal of 500 kg

^b^Stocking rates expressed in livestock units ha^−1^ year^−1^

### Dung and vegetation nitrogen measurements

During the grazing seasons of 2019 and 2020, eight to 12 fresh dung pats of different Highland cows were sampled, two to three times (period 1, 2 and 3) in every paddock, every 10 days, around noon. Before analyses, all dung samples were freeze-dried (Christ Delta 2–24, Kühner AG, Birsdelden, Switzerland) and milled through a 1.0 mm sieve (Brabender rotary mill; Brabender GmbH & Co. KG, Duisburg, Germany). Nitrogen (N) content was determined by the Dumas method (ISO 16634-1:2008). To determine residual dry matter content, samples were dried during 3 h at 105 °C.

Three samples of *A. viridis* leaves (1750 g of fresh leaves each) were collected in both sites of Bovonne and Val Vogna, in June, July and August of both years to represent the changes in leaf N content during the summer season. For each sample, *A. viridis* leaves (including petioles) were hand-harvested all around the canopy of five different trees up at a maximum above-ground height of 1.80 m to simulate the potential grazing by Highland cattle (Svensk et al. 2022). In each paddock, 12 to 66 vegetation patches of 0.47 ± 0.05 ha (0.01 ha to 1.7 ha) were defined prior to Highland cattle grazing, representing areas with homogeneous botanical composition and vegetation structure. Herbaceous vegetation samples (250 g of fresh matter) were then sampled before grazing using a handheld grass cutter in each vegetation patch (one vegetation sample per patch) in 2019 for paddocks 1, 2, and 3, and in 2020 for paddock 4. Leaf and herbaceous vegetation samples were dried at 60 °C for 72 h. After being ground to pass a 1-mm sieve (Brabender rotary mill; Brabender GmbH & Co. KG, Duisburg, Germany), leaf and herbaceous vegetation samples were analysed for dry matter content by heating at 105 °C during 3 h. The N content of *A. viridis* leaves and herbaceous vegetation samples was determined by the Dumas method (ISO 16634-1:2008). Moreover, in each vegetation patch the percentage of *A. viridis* cover was also assessed using direct visual observations and satellite pictures, and the slope was calculated through a Digital Terrain Model (90-m resolution) in QGIS 3.6 software.

### Nitrogen ingested estimation

The weighted mean of N ingested by Highland cattle was estimated during a period of 24 h before dung sampling (N_24H_), following the methodology on cattle diet timespan estimations (Estermann et al. 2001; Bakker et al. 2004; Browne et al. 2005). For this 24 h timespan, Highland cattle activities (grazing and resting) were discriminated through the analysis of both horizontal distance travelled and activity data from the GPS collars sensors, considering that Highland cattle were ingesting N during the grazing activity phases only. The horizontal distance travelled was calculated from consecutive GPS fixes for each collared cow. The activity from collar sensors was obtained from the mean of X and Y axes activities. Therefore, we identified the phases when grazing occurred most frequently. Grazing phases were identified as times when distance travelled and motion sensor-based activity were higher, and resting phases were assigned times when these values were lower (Probo et al. 2014). The number of GPS fixes within the grazing activity phases was then counted in each vegetation patch. Then, the proportion of GPS fixes in each vegetation patch was related to the measured herbaceous vegetation N content of the patches, to compute the weighted mean of N ingested over the 24 h (N_24H_) according to the following formula:

1 $${N}_{24H}= \frac{\sum_{i}^{n}\left({NHV}_{i}*{GPSga\;fixes}_{i}\right)}{\sum_{i}^{n}{GPSga\;fixes}_{i}}$$

where NHV_i_ and GPSga fixes_i_ represent the N content of the herbaceous vegetation and the proportion of GPS fixes within the grazing activity time in the vegetation patch i, 24 h before dung sampling, respectively.

In addition, in all patches where *A. viridis* was present, the N_24H_ calculated from herbaceous vegetation was corrected based on the N content of *A. viridis* leaves sampled at the closest date to the corresponding dung sample, to account for the N ingested from *A. viridis* leaf consumption. Based on the direct observations of Highland cattle foraging behavior conducted in Bovonne (Nota et al. 2022, 1077 observations), it was estimated that *A. viridis* leaves represented 12% of animal diet on average (although we also incorporated variation in the amount of *A. viridis* leaves eaten, see below). Thus, in the vegetation patches in which *A. viridis* was present, the N_24H_ corresponding to each dung sample was calculated considering a diet characterized by 88% N from herbaceous forage and 12% N from leaves, following this formula:

2 $${N}_{24H}= \frac{\sum_{i}^{n}\left[\left[\left(0.88*{NHV}_{i} \right)+\left({0.12*NAL}_{i}\right)\right]*{GPSga\;fixes}_{i}\right]}{\sum_{i}^{n}{GPSga\;fixes}_{i}}$$

where NAL_i_ represents the N content of *A. viridis* leaves sampled at the closest date to the corresponding dung sample.

### Nitrogen import–export flux estimation

In every paddock and for each vegetation patch, the value of the N flux (N kg ha^−1^ yr^−1^) was calculated using an import–export model following this formula:

3 $$Nflux=Nexcreted-Ningested$$

where Nexcreted is the estimated amount of N excreted by livestock through urine and dung in the patch during the whole grazing season, and Ningested is the estimated amount of N eaten by livestock in the same patch and during the same period.

For this flux, two assumptions were made: (i) the excretion is proportional to the total time cows spent in the patch, and (ii) the ingestion is proportional to the time cows spent grazing only (White et al. 2001; Koch et al. 2018).

Based on these assumptions, the excreted N was calculated as:

4 $$Nexcreted = {\text{D}}*{\text{TS}}*{\text{n}}*{\text{DM}}*\left( {{\text{p}}*{\text{Npaddock}}} \right)$$

where D is the number of grazing days, TS the percentage of time spent by cows in the vegetation patch, n the number of animals present, DM the dry matter intake (kg animal^−1^ day^−1^) calculated using previous studies on Highland cattle weight and DM ingestion of both cows and calves (Berry et al. 2002; Pauler et al. 2019), and Npaddock the estimated weighed mean of N eaten by the cows (g kg^−1^ DM) in the whole paddock, using the N content of *A. viridis* leaves and herbaceous vegetation as previously described. The parameter p is the estimated percentage of Ningested that is excreted by grazing beef cattle according to seven trials conducted in comparable conditions and presented in Estermann et al. (2003), Berry et al. (2002) and Estermann et al. (2001). Indeed, those experiments had comparable conditions to our study as they were performed on low growing beef cattle breeds (Highland or Angus cattle with cow and calf pairs), in similar montane environments (1557 ± 237 m) and/or with similar crude protein content diets (14.63 ± 0.75%, compared to 16.05 ± 1.12% from our N ingested estimations).

Ingested N was calculated as:

5 $$Ningested = {\text{D}}*{\text{TG}}*{\text{n}}*{\text{DM}}*{\text{Npatch}}$$

where TG is the percentage of time cows spent grazing in the patch, and Npatch the estimated ingested N content calculated from the N content of *A. viridis* leaves and herbaceous vegetation, at the vegetation patch level.

Since some parameters from the estimation of the Nbalance are subjected to uncertainties, we associated a statistical distribution to each parameter (mean ± SE), following the methodology described in Koch et al. (2018), and performed 500 Monte Carlo simulations, in order to obtain 5%, 50% and 95% quantiles of the N flux for each vegetation patch in every year. We thus included the uncertainty of: (i) p from the seven experiments (92.7 ± 0.7%), (ii) Npaddock, by including the variability of the percentages of *A. viridis* leaves eaten by the cows when in presence of the shrub (11.8 ± 2.8%), at the paddock level, and (iii) Npatch, by including the same variability of the percentages of *A. viridis* leaves eaten by the cows when in presence of the shrub at the patch level.

### Statistical analysis

All statistical analyses were performed using R version 3.4.4 (R Core Team 2017). The effect of *A. viridis* cover on the N content of the understory herbaceous vegetation was tested by using a Generalized Least Square model (GLS), with *A. viridis* cover as fixed factor and coordinates of vegetation patches centroids nested into “paddock” as random effect, using a Linear Correlation Structure. This random effect structure accounts for the nested structure of the data and for any residual spatial autocorrelation among neighboring vegetation patches.

The relation between dung pats N content (response variable) and N_24H_ (explanatory variable) was tested using a Linear Mixed-effect Model (lme, package “nlme”), with the date of dung sampling as a continuous variable nested into “paddock” as a time correlated covariate (corCAR1), to account for the temporal autocorrelation structure linked to vegetation changes throughout the summer season. The marginal R-squared values were obtained using the “performance” package (function “model_performance”).

The effect of *A. viridis* cover (divided into 3 categories: 0–33%, 34–66% and 67–100%), slope (divided into 3 categories: < 10°, 10–20° and > 20°) and their interaction on the N fluxes was tested using a Linear Mixed-effect Model lme, package (“nlme”). Paddock was specified as random factor to account for spatial autocorrelation. Model residuals were affected by heteroscedasticity, therefore a weighting function was used to correct the variances through the argument *varIdent* in the *lme* function by setting as grouping variable the *A. viridis* covers. Post hoc tests were performed for the models when significant effects between categories were detected (Tukey’s test, *P* < 0.05, emmeans package).

## Results

### Effect of *A. viridis* cover on the nitrogen content of the understory herbaceous vegetation

**Fig. 1:**
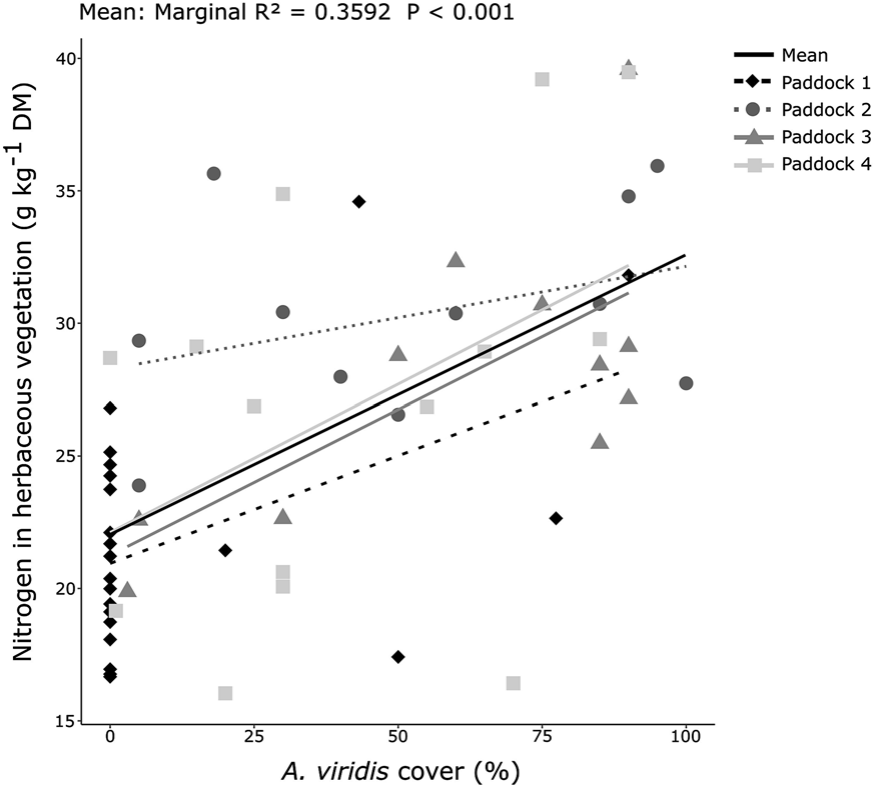
Relationship between the N content of the understory herbaceous vegetation and *A. viridis* cover percentage in the vegetation patches of all paddocks

The herbaceous vegetation had an average N content of 25.9 ± 0.8 g kg^−1^ DM among all paddocks and vegetation patches (the details per paddock can be found in supplementary materials S1–S4). The N content of the herbaceous vegetation was positively related with *A. viridis* cover percentage (Fig. 1, *P* < 0.001, R^2^ = 0.36, n = 58). Indeed, the N concentration of the herbaceous vegetation increased with increasing *A. viridis* cover, with averages of 22.9 ± 0.9 g kg^−1^ DM in open areas (0–33% of *A. viridis* cover), 28.2 ± 1.6 g kg^−1^ DM in moderately encroached areas (34–66% of *A. viridis* cover) and 30.5 ± 1.6 g kg^−1^ DM in highly encroached areas (67–100% of *A. viridis* cover).

### Effect of animal diet on dung nitrogen content

**Fig. 2:**
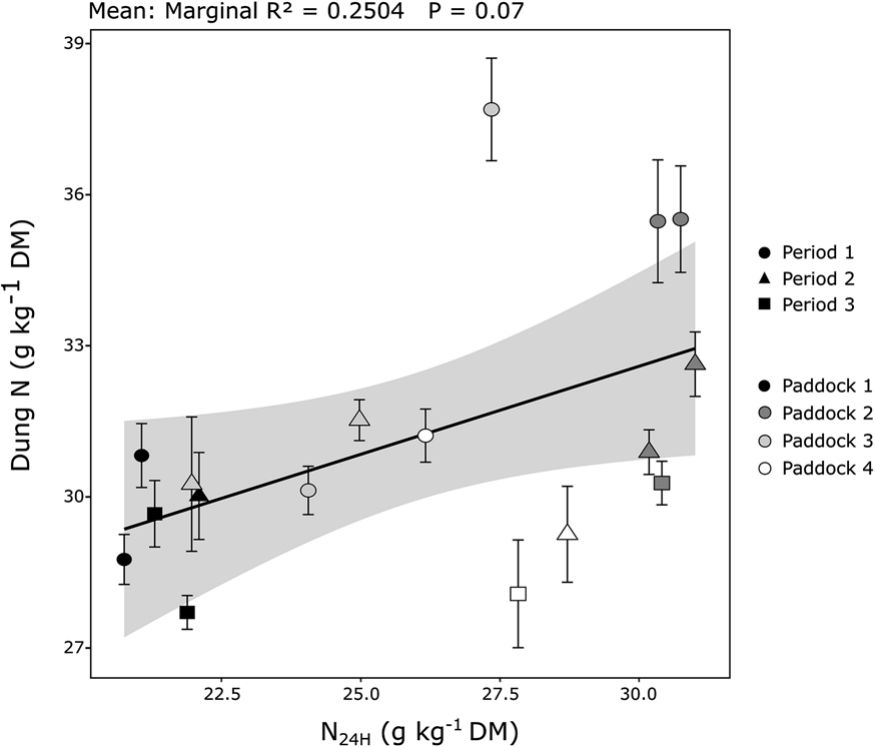
Relationship between the N content of the dung pats (dung N, g kg^−1^ DM) of Highland cattle and the estimated mean content of N ingested 24 h before the dung sampling (N_24H,_ g kg^−1^ DM), with the trend line (grey area) following lm smoothing method, and error bars representing the standard error

The N content of Highland cattle dung pats was on average 31.2 ± 0.2 g kg^−1^ DM (Fig. 2, mean ± SE), and consistent between both years (31.1 ± 0.3 g kg^−1^ DM in 2019, and 31.4 ± 0.4 g kg^−1^ DM in 2020, *P* = 0.137). The N dung content slightly decreased during the grazing season in both years (*P* < 0.001), with averages of 32.8 ± 0.5 g kg^−1^ DM, 31.0 ± 0.3 g kg^−1^ DM and 29.2 ± 0.4 g kg^−1^ DM in dung sample periods 1, 2 and 3 respectively.

The N content of *A. viridis* leaves that was used for the estimation of the N_24H_ of the encroached vegetation patches was of 31.8 ± 0.6 g kg^−1^ DM across all sites and periods. The estimated N_24H_ remained stable across dung sampling periods (*P* = 0.92), with 25.8 ± 1.5 g kg^−1^ DM, 26.5 ± 1.6 g kg^−1^ DM and 25.4 ± 2.2 g kg^−1^ DM in period 1, 2 and 3 respectively. The N_24H_ ingested was of 21.4 ± 0.3 g kg^−1^ DM, 30.5 ± 0.2 g kg^−1^ DM, 24.6 ± 1.1 g kg^−1^ DM and 27.6 ± 0.8 g kg^−1^ DM for paddock 1, 2, 3 and 4 respectively, with an overall average of 25.9 ± 1.0 g kg^−1^ DM among all paddocks. There was a marginally significant positive relationship between dung N content and N_24H_, (Fig. 2, *P* = 0.07, R^2^ = 0.25, n = 17).

### N import–export fluxes in vegetation patches

The 5%, 50% and 95% quantiles of the predicted values displayed similar patterns, for N ingested, N excreted and the resulting N fluxes (Table 2). The following results are values of the 50% quantile (see Maps of N fluxes per each paddock in Supplementary materials S5–S9).

**Table 2:**
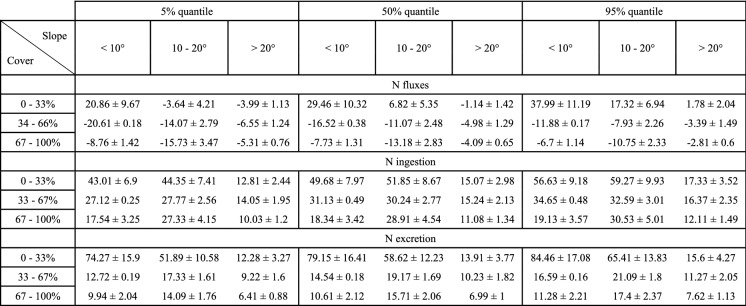
Mean ± SE of the N fluxes, and detailed N ingestion and N excretion (kg ha^−1^ yr^−1^) values for 5%, 50% and 95% quantiles, estimated on vegetation patches of all paddocks on both years, depending on slope and *A. viridis*-cover categories

**Fig. 3:**
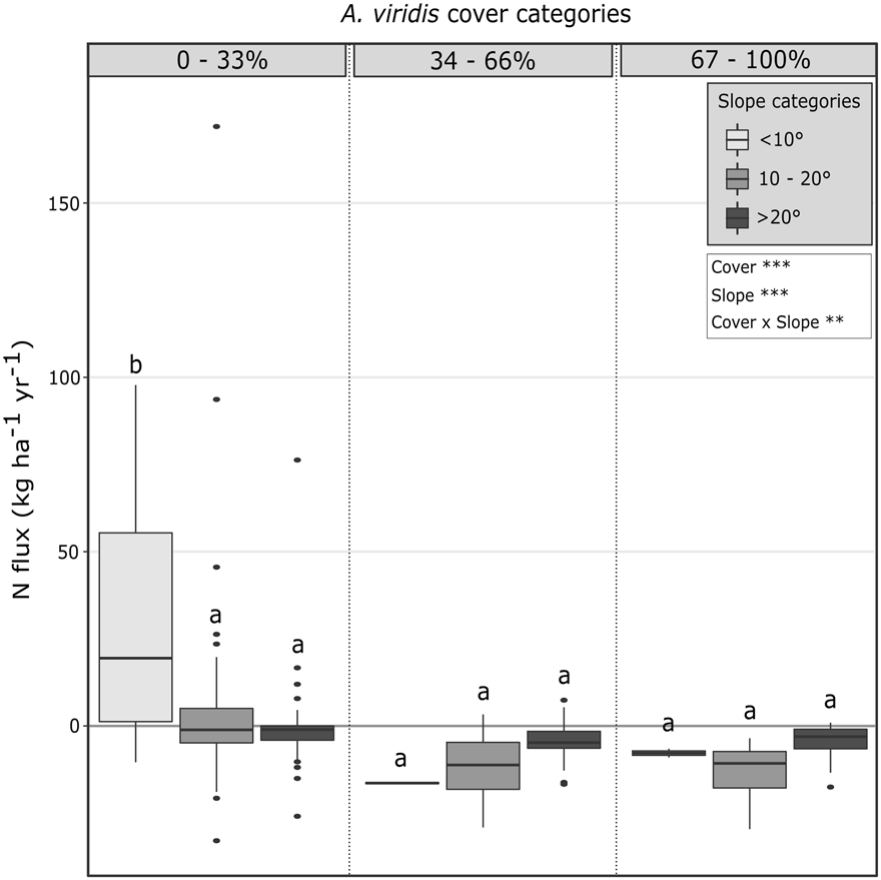
N fluxes (kg ha^−1^ yr^−1^) of all paddocks and both years for the different categories of *A. viridis* cover (0–33%; 34–66%; 67–100%) and slope (< 10°; 10–20°; > 20°). Different letters indicate significant differences between categories

Based on N fluxes, in all paddocks, Highland cattle visited and actively grazed mostly on the open and moderately flat areas. Indeed, the most open vegetation patches (0–33% of *A. viridis* cover) with medium slope (10–20°) displayed the highest mean N ingestion, i.e. 51.9 ± 8.7 kg ha^−1^ yr^−1^. On the other hand, the steepest (> 20°) and most encroached areas (67–100%) displayed the lowest average of N ingested, i.e. 11.1 ± 1.3 kg ha^−1^ yr^−1^ (Table 2). In parallel, N excretion was the highest in the open areas (0–33%) and in the lowest slopes (< 10°), with a N excreted average of 79.2 ± 16.4 kg ha^−1^ yr^−1^. Similar to N ingestion, the lowest N excretions were estimated in steepest and most encroached areas, with a N excretion value of 7.0 ± 1.0 kg ha^−1^ yr^−1^. Consequently, the N fluxes were significantly affected by *A. viridis* cover and slope (Fig. 3, *P* < 0.001), with overall positive N fluxes in flat and open areas (i.e. N accumulated), and negative N fluxes in steeper and encroached ones (i.e. N exported; Fig. 3, Table 2). On average, open and flat areas had N fluxes of 29.5 ± 10.3 kg ha^−1^ yr^−1^, while encroached and steep areas had average N fluxes of -4.1 ± 0.7 kg ha^−1^ yr^−1^. Indeed, in all paddocks combined, 80.66% of vegetation patches with positive N fluxes were open pastures (0–33% of *A. viridis* cover), while only 2.2% were highly encroached areas (67–100% of *A. viridis* cover), and 17.14% were moderately encroaches areas (34–66% of *A. viridis* cover). Most (44.81%) of these accumulation zones had a moderate slope (17.5 ± 0.6° on average). On the other hand, 37.9% of the vegetation patches that displayed a N depletion were open pastures, 47.08% were highly encroached areas and 15.05% were moderately encroached areas. Most (74.0%) of these depletion zones had high slope (30.5 ± 0.6° on average). Moreover, in terms of spatial distribution, N accumulated in very small areas and most of the grazed land was N depleted. Indeed, paddocks 1, 2, 3 and 4 displayed N accumulation in only 11.8%, 15.1%, 12.1% and 19.2% of their areas, respectively.

N fluxes were very similar among years. Indeed, vegetation patches with negative N fluxes had on average − 6.9 ± 0.8 kg ha^−1^ yr^−1^ in 2019, and − 5.6 ± 0.7 kg ha^−1^ yr^−1^ in 2020, while vegetation patches with positive N fluxes had an average of 26.0 ± 10.3 kg ha^−1^ yr^−1^ in 2019, and 19.3 ± 5.7 kg ha^−1^ yr^−1^ in 2020.

In 2019, four vegetation patches were not visited by Highland cattle in paddock 1, corresponding to 0.98% of the paddock size (0.30 ha). In 2020, three of these patches remained unvisited with an addition of two others, corresponding to 1.34% of the paddock size (0.41 ha). As a result, estimations of N ingested, N excreted and N fluxes were equal to zero for those unvisited areas. Most of these vegetation patches were covered by *Rhododendron ferrugineum* or were *A. viridis*-encroached areas, and one was an open pasture with steep slope (34.8°). All vegetation patches of paddocks 2, 3 and 4 were visited in both years.

## Discussion

### *A. viridis* cover increases the nitrogen content of the understory herbaceous vegetation

In all paddocks of the study, the N content of herbaceous vegetation samples was significantly higher in the understory of *A. viridis* stands than in adjacent open pastures, as previously observed by Bühlmann et al. (2016), Zehnder et al. (2016, 2020), and Svensk et al. (2021). Bühlmann et al. (2016) also demonstrated that the N content in herbaceous plant leaves under *A. viridis* canopy was as high as the N content in *A. viridis* leaves. Indeed, they measured similar concentrations in plant leaves growing in the understory of *A. viridis* shrublands, with values between 27.3 ± 4.5 g kg^−1^ DM and 30.7 ± 3.3 g kg^−1^ (at 1650 and 1950 m elevation respectively), similar to our mean concentration of 30.5 ± 1.6 g kg^−1^ DM g kg^−1^ measured in highly encroached areas. Therefore, *A. viridis* shrublands accumulate N in the ground soil and provide an ideal habitat for nitrophilous plants with high N concentrations in their leaves (Anthelme et al. 2001; Bühlmann et al. 2016; Zehnder et al. 2020). Moreover, because *Alnus* species do not need to mobilize N from their leaves in autumn (Rodríguez-Barrueco et al. 1984), they resorb very little of their leaf N content. Combined with the late shading of their leaves, this leads to high N contents in the litter and to increased soil N saturation over time (Bühlmann et al. 2016).

### Animal diet affects dung nitrogen content

The N content measured in Highland cattle dung pats was high, with an average of 31.2 ± 0.2 g kg^−1^ DM, if compared to values found for other cattle breeds with high crude protein rich diets, thus confirming our first hypothesis. For instance, two studies conducted by Koenig and Beauchemin (2013a, b) found that Angus beef cattle fed with silage of similar crude protein content (14–14.5%) excreted a fecal N of about 21.2 g kg^−1^ DM to 23.2 g kg^−1^ DM on average. Haynes and Williams (1993) determined that the average N content in the dung of dairy cattle grazing in open pastures varied between 20 and 28 g kg^−1^ DM, whereas the N dung concentration was about 27 g kg^−1^ DM in nutrient-rich pastures (Williams and Haynes 1995). Similarly, Lançon (1978) found a dung N content of 20.6 g kg^−1^ DM for non-fertilized grasslands and 28.7 g kg^−1^ DM for fertilized grasslands. Other studies have also assessed dung N contents around 20 g kg^−1^ DM for dairy cows grazing on open pastures (Yokoyama et al. 1991; Bakker et al. 2004).

The high dung N content found in our study was marginally related to the amount of N ingested during 24 h before excretion, showing a trend of increasing N in the dung with increasing ingested N, which is in line with our second hypothesis. Despite the non-significance of this effect, the trend shown in this relation suggests that the more Highland cattle grazed in highly encroached areas, where N content in vegetation was high, the richer their dung became in N. This result is in line with previous studies, such as Kebreab et al. (2001) and Angelidis et al. (2019). For example, by using data of 69 different studies, Angelidis et al. (2019) showed that the N intake had a significant positive effect on the excreted dung N. While dung N seemed to increase with increasing N_24h_, even the lowest dung N concentrations recorded in this study remained rather high compared to other experiments in similar conditions. This might be mainly explained by the overall high N content in the diet of Highland cattle grazing in these *A. viridis*-encroached areas, The high N content in dung pats may also be explained by the high condensed tannin concentration of *A. viridis* leaves. Indeed, Stević et al. (2010) demonstrated that *A. viridis* leaves can have a tannin concentration of 44 ± 4 g kg^−1^ DM, which can form tannin-protein complexes by binding with plant proteins when consumed by livestock (Harris et al. 1998; Waghorn 2008; Woodfield et al. 2019). These complexes are better protected from rumen microbial degradation and they can thus reduce the degradation of N during digestion (Waghorn et al. 1987; Burggraaf and Snow 2010; Piñeiro-Vázquez et al. 2017). Therefore, the passage of undegraded N could be favored through the intestine and through dung, and a smaller proportion of N might be excreted in urine (Burggraaf and Snow 2010; Theodoridou et al. 2011). Further studies on Highland cattle digestion are needed to confirm whereas the proportion of N excreted in urine will be reduced through the consumption of *A. viridis*-encroached vegetation with high tannins concentrations, as this could be beneficial. Indeed, a reduction of urine N may decrease N pollution, as urea N is subjected to ammonia (NH_3_) volatilization and nitrate leaching (Woodmansee et al. 1981; Jarvis 1994; Whitehead 1995; Tamminga 2006; Angelidis et al. 2019). On the other hand, NH_3_ volatilization through the dungs is restrained by the crust formation of dung pats, even though heavier rainfall might alter this protection (Longhini et al. 2020). Fecal N is in a less mobile form that needs mineralization before N can be lost through leaching, and NH_3_ volatilization through urine is 5–6 times higher than through dung pats (Lockyer and Whitehead 1990; Kebreab et al. 2001; Berry et al. 2002; Woodfield et al. 2019). Furthermore, the slow process of N release through dung pats can allow a better use by the surrounding vegetation and for the soil on the long term (Woodmansee et al. 1981; Lantinga et al. 1987; Berry et al. 2001; Burggraaf and Snow 2010). Moreover, the excretion of condensed tannins through dung may also inhibit nitrification and slow down the microbial activity of the soil, preventing nitrate leaching and consequently soil acidification (Burggraaf and Snow 2010). Finally, requirements for cattle diet show that a minimum of 20 to 40 g kg^−1^ DM of condensed tannin concentration is needed to improve animal performance (Woodfield et al. 2019), which, combined with the N supply *A. viridis* leaves provide, highlight their forage potential for robust breeds, such as Highland cattle. However, further research is needed to assess the *A. viridis* leaf ingestion by grazing cattle on a higher range of environmental conditions.

### An active nitrogen translocation occurred from shrub-encroached areas to pastures

Our study demonstrated that the excretion of N after its uptake from *A. viridis* leaves and the understory herbaceous vegetation allowed its redistribution across the pastures. Overall, the N transfer was significantly dependent on *A. viridis* cover as well as on topographic features (i.e. slope), confirming our third hypothesis. Indeed, our estimations of the N fluxes in the paddocks showed that there was an active N translocation from the steep, shrub-encroached, and N saturated areas to the adjacent flat and open pastures that had comparatively lower herbaceous N content. This is consistent with the findings of Schnyder et al. (2010), who showed a significant N accumulation in the flattest zones and N removal from the steepest areas in hilly pastures. In the same study, the authors found similar results for phosphorus (P), which is a good estimate of other nutrients such as N, with the difference that this element is much less present in urine and thus easier to evaluate on field and less subjected to transformations. Similar results on P were found in previous studies, such as Koch et al. (2018), where P was significantly translocated from feeding areas to the flattest resting areas, and Jewell et al. (2007), who also found a significant redistribution and fewer dung deposition in the steepest areas of subalpine pastures. As in previous studies, our flat and open areas with high N deposition often correspond to livestock resting areas, where there is generally a high excretion deposition, especially during the night. Costa et al. (1990) found that 90% of cattle excretion was deposited in resting-adapted areas, with slopes lower than 40%. In the present study, while the open areas displayed the highest N ingestion as well as the highest N excretion, the latter often exceeded N ingestion, making the final N fluxes positive. In the same way, the highly encroached areas displayed the lowest N ingestion, but displayed an even lower N excretion, highlighting the final depletion of N induced by Highland cattle grazing. On the other hand, the depletion areas of our paddocks were larger than the accumulation zones, meaning that cattle actively removed N from a larger area and concentrated it in relatively small patches. This is in line with previous findings, which showed that nutrients are often returned into small spots, with most of the area displaying a negative balance and net nutrient loss (Bakker et al. 2004; Jewell et al. 2007; Koch et al. 2018).

The assessment of the N fluxes over two years allows us to suggest that Highland cattle grazing could moderately reduce N accumulation under *A. viridis* encroached stands on the long-term, while simultaneously providing it in targeted areas. Particularly, the proper management of resting areas could help fertilize specific nutrient-poor pastures which are subjected to early encroachment processes (Probo et al. 2016), such as pastures dominated by *Nardus stricta* L., *Rhododendron ferrugineum* L. or *Vaccinium myrtillus* L. The translocation of N towards these areas could improve their forage yield and quality, potentially transforming the N saturation issue into an environmental and agronomical resource. Schellberg et al. (1999) demonstrated in a long-term study that N and P inputs on nutrient-poor grassland drastically increased DM production and forage quality and changed floristic composition. In this study, oligotrophic species decreased with nutrient fertilization, and created ecological favorable conditions for other grasses and legumes. At a moderate level, fertilization can improve botanical diversity (Vintu et al. 2011) and, with N and P enrichment by Highland cattle grazing, we could expect similar results on the long-term whether such management should continue. However, further studies, not only on N import–export fluxes related to grazing, but also on N pool changes are needed to better understand the potential of nutrients translocation, and the benefits for the vegetation and soil characteristics. Indeed, N inputs from atmospheric deposition and N fixation can be quite elevated in these environments (50 to 100 kg ha^−1^ yr^−1^ according to Binkley et al. 1994). Despite our results showing moderate N translocation towards open and flat areas (29.52 kg DM ha^−1^ yr^−1^ on average), attention should be paid to possible over-fertilization issues resulting from high levels of N accumulation in the resting areas. Indeed, the distribution patterns of grazing cattle can be stable across years (Koch et al. 2018), consequently leading to a repeated distribution of cattle dung and thus potential over-fertilization of the resting areas. To avoid such negative impacts and preserve nutrient capital, it is advised to adopt a rotational management, as it allows more homogeneous utilization of the pasture compared to free-grazing systems. Previous studies have shown that the presence of fences, shaded zones, attractive points (such as salty or molasses blocks), and a water trough may also impact the spatial distribution of dung and urine pats (Jewell et al. 2007; Buttler et al. 2008; Auerswald et al. 2010; Pittarello et al. 2016; Carnevalli et al. 2019; Svensk et al. 2022). Thus, these features should be included in the management of cattle grazing to better redistribute nutrients across the pastures. The results of this study highlight the potential of Highland cattle grazing to become an efficient tool to effectively translocate part of the ingested N, which could be accounted for in livestock management, and help in the restoration of former open pastures in the long-term. However additional research is needed to assess the effect of such grazing management to counteract N deposition in the shrublands, including long term effects on nutrients pools.

## Supplementary Information

Below is the link to the electronic supplementary material.

Supplementary file1 (DOCX 17921 KB)
