# Supplementary material for: Nitrogen translocation by Highland cattle grazing in Alnus viridis-encroached pastures
Source: Nutr Cycl Agroecosyst. 2023 Apr 10;126(1):127–41. doi: 10.1007/s10705-023-10282-0 (PMC10129924; doi:10.1007/s10705-023-10282-0)

**Supplementary S1** – Nitrogen content (g kg^-1^ DM) of the herbaceous vegetation and *Alnus viridis* cover (%) of all vegetation patches of paddock 1.


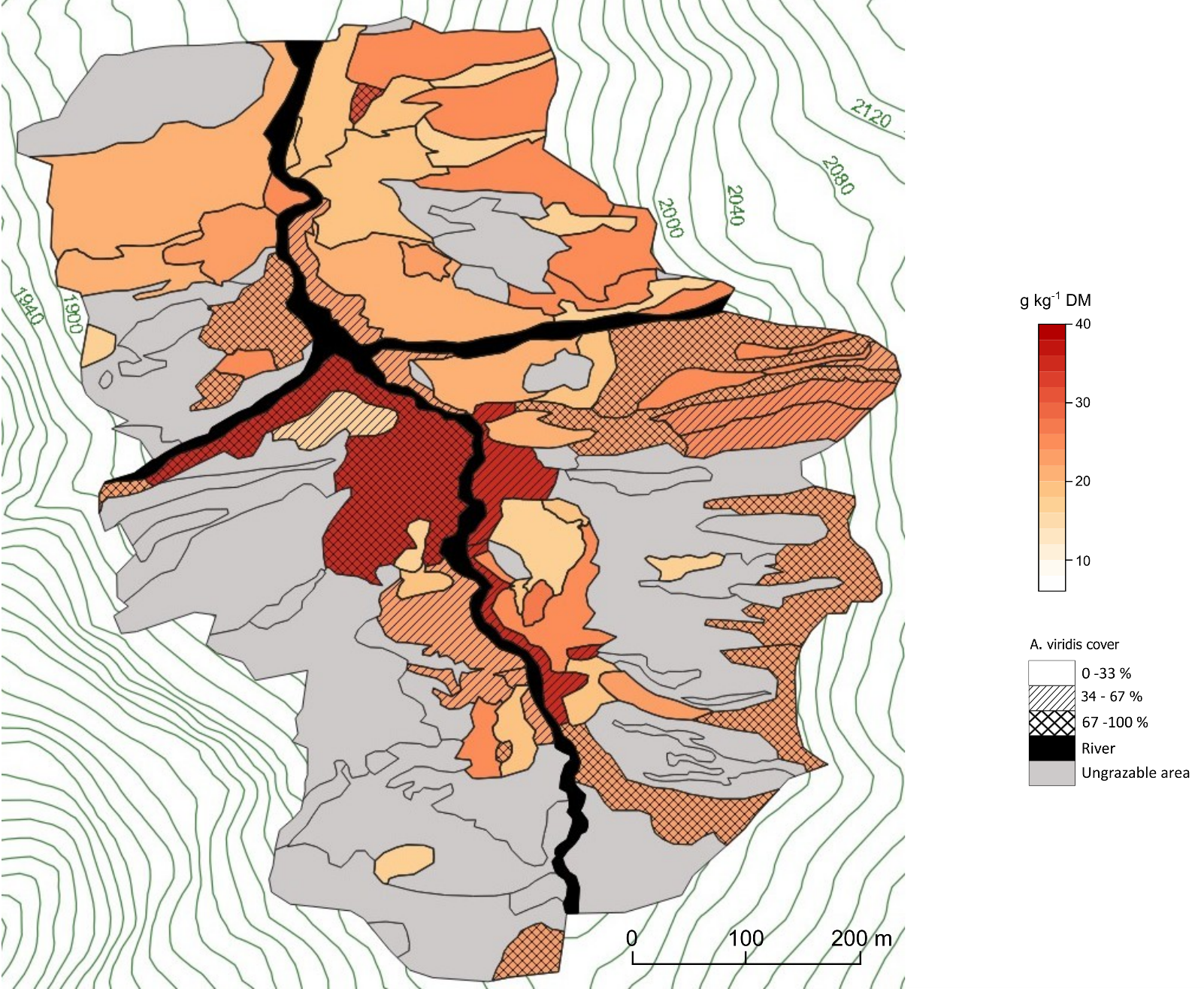


**Supplementary S2** – Nitrogen content (g kg^-1^) of the herbaceous vegetation and *Alnus viridis* cover (%) of all vegetation patches of paddock 2.


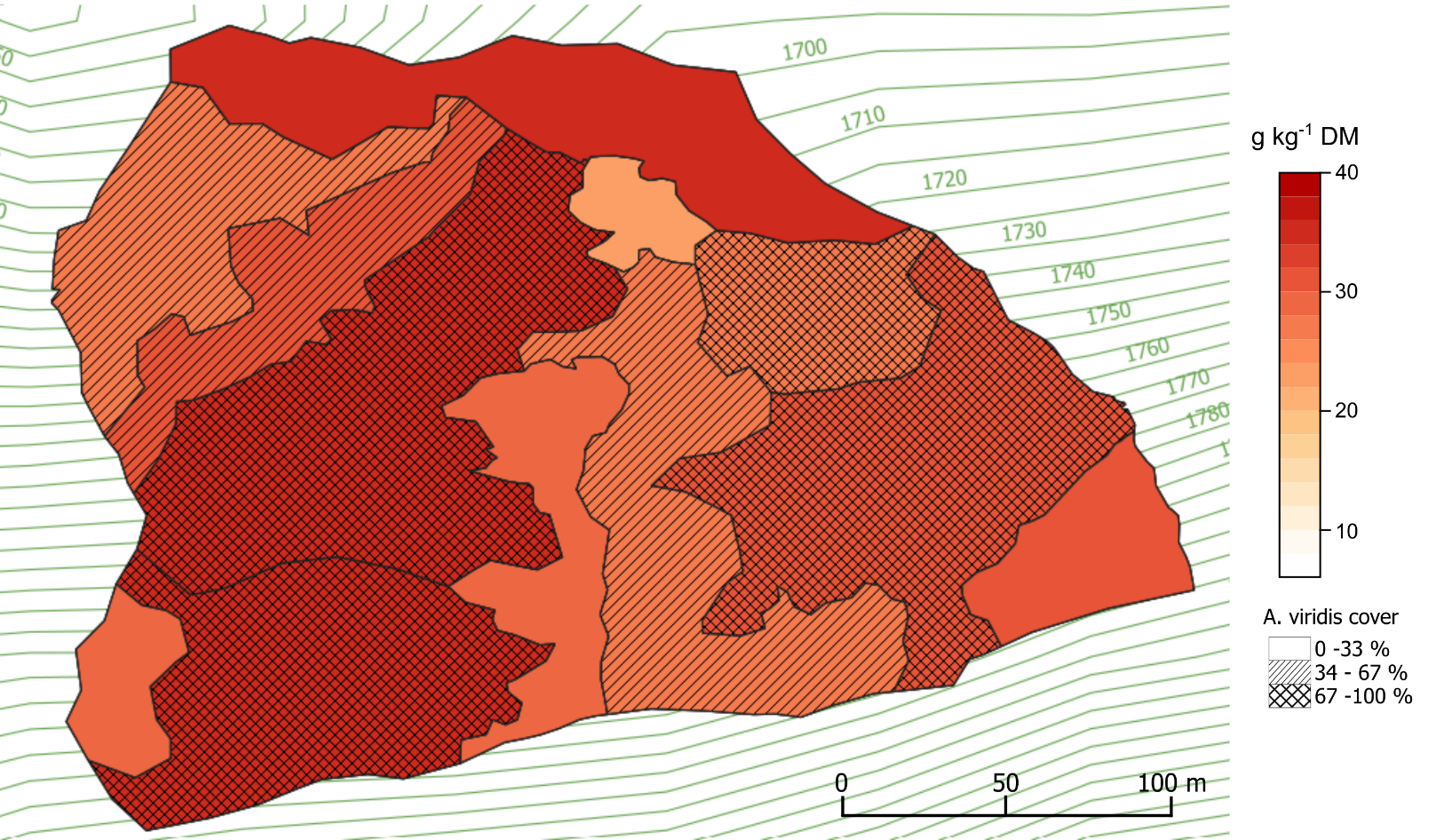


**Supplementary S3** – Nitrogen content (g kg^-1^) of the herbaceous vegetation and *Alnus viridis* cover (%) of all vegetation patches of paddock 3.


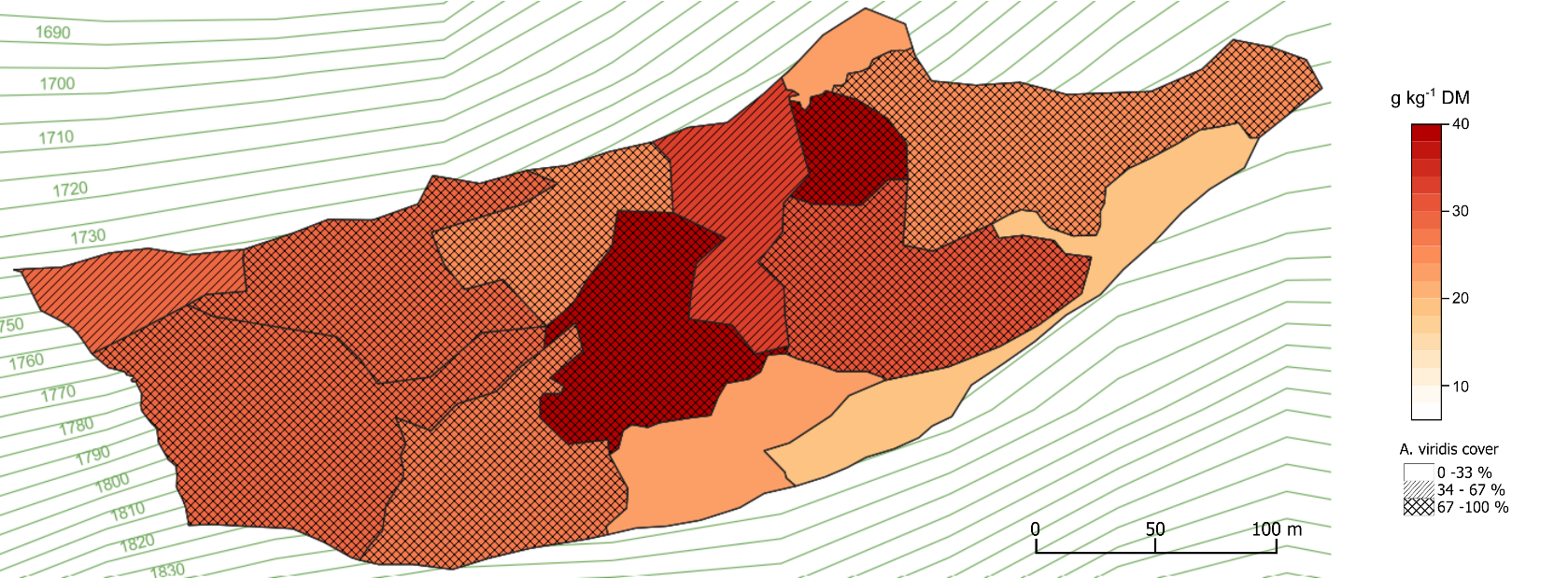


**Supplementary S4** – Nitrogen content (g kg^-1^) of the herbaceous vegetation and *Alnus viridis* cover (%) of all vegetation patches of paddock 4.


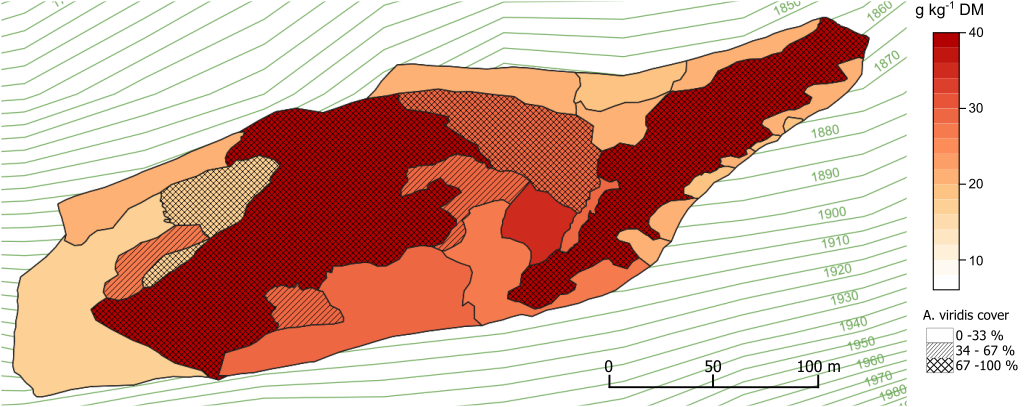


**Supplementary S5** –Nitrogen fluxes (50% quantile, kg ha^-1^ yr^-1^) of the vegetation patches of paddock 1 in 2019.


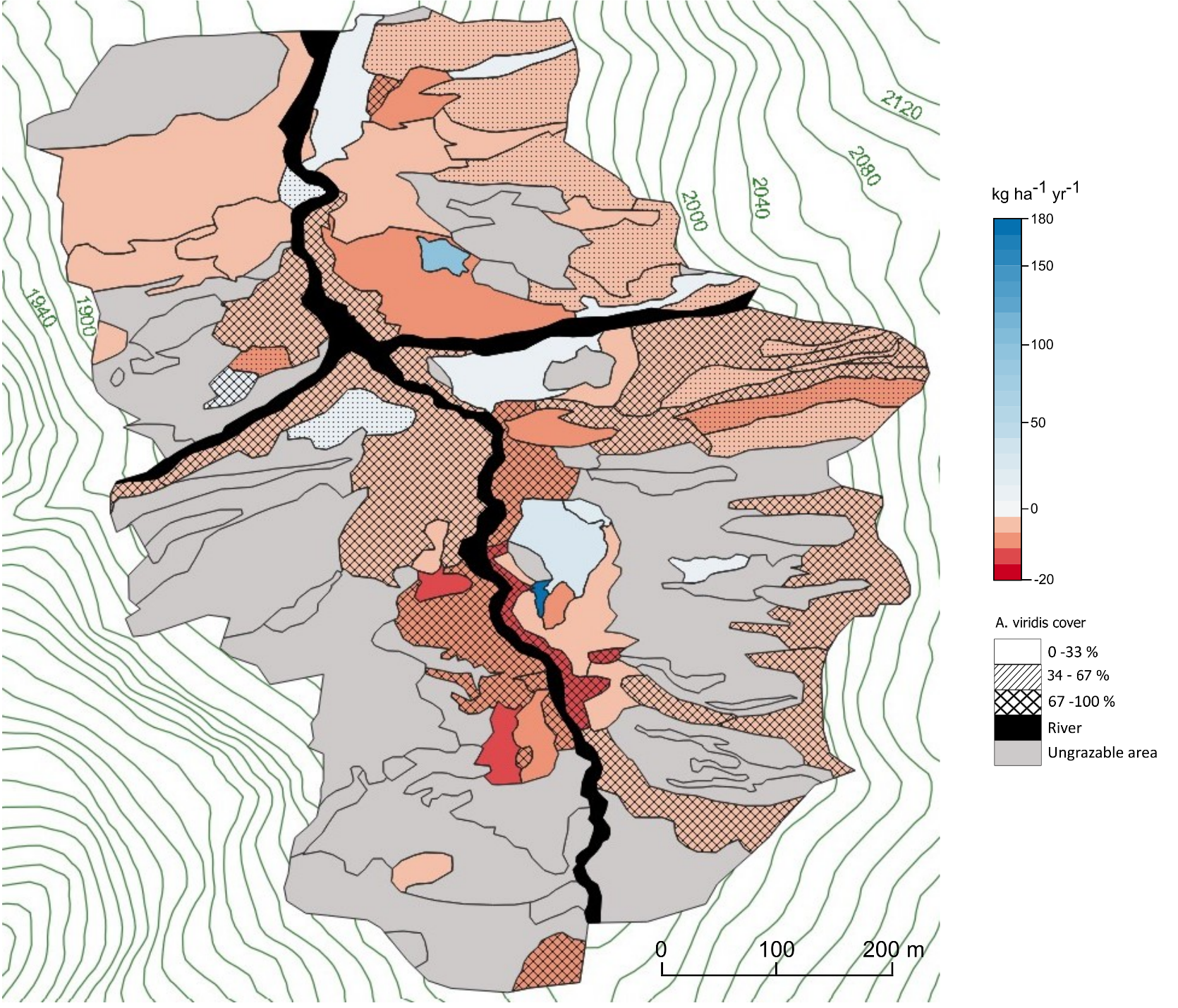


**Supplementary S6** –Nitrogen fluxes (50% quantile, kg ha^-1^ yr^-1^) of the vegetation patches of paddock 1 in 2020.


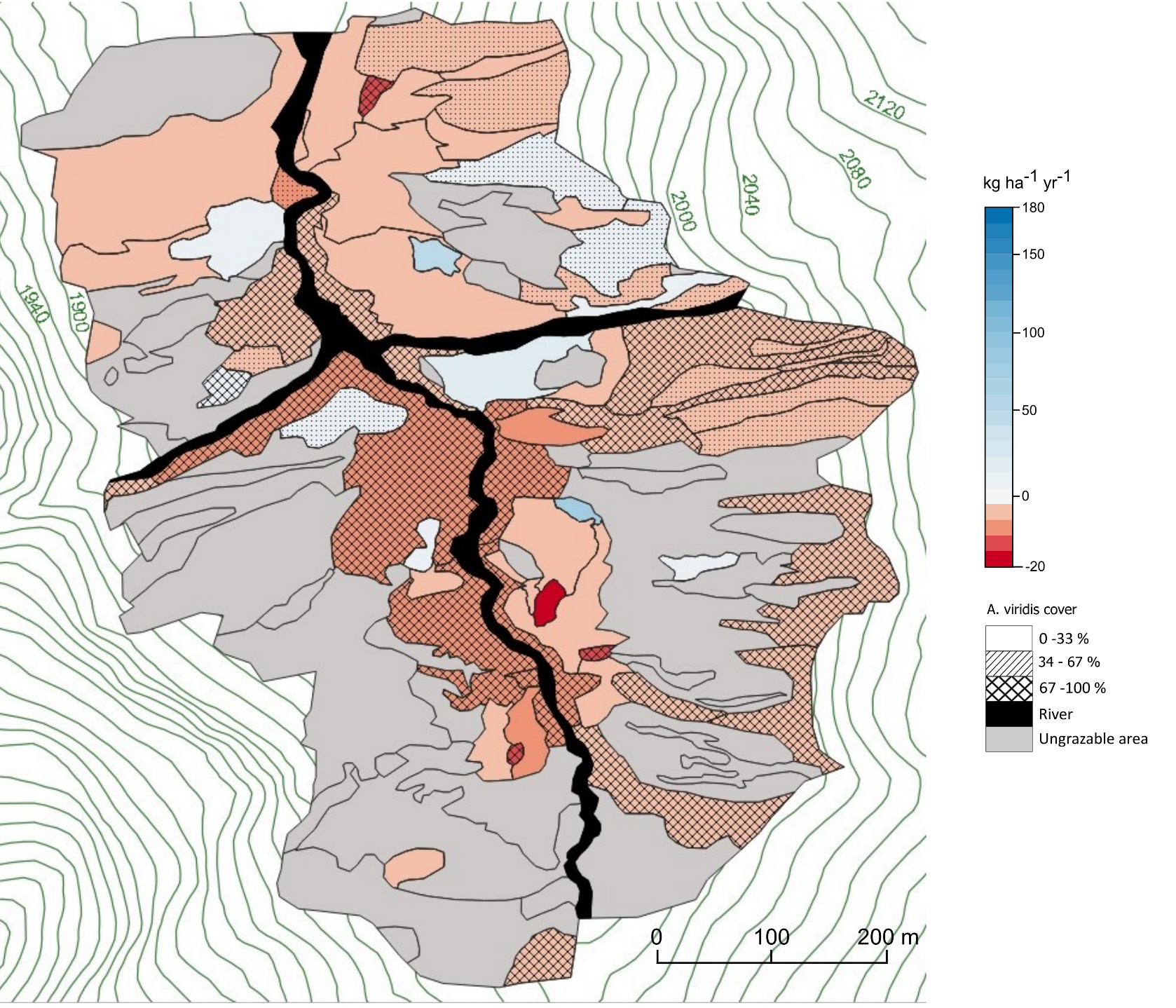


**Supplementary S7** –Nitrogen fluxes (50% quantile, kg ha^-1^ yr^-1^) of the vegetation patches of paddock 2 in: a) 2019, and b) 2020.


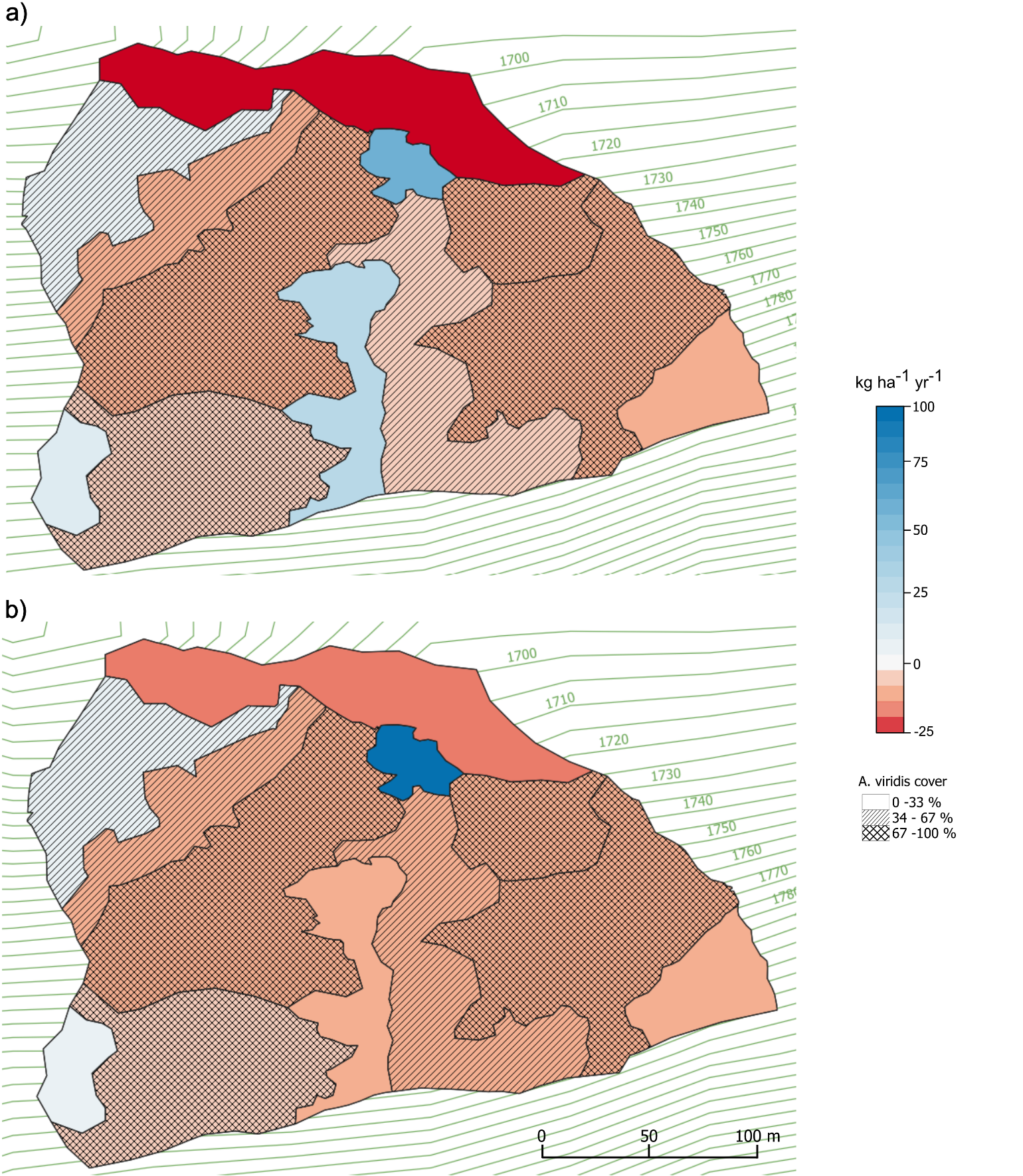


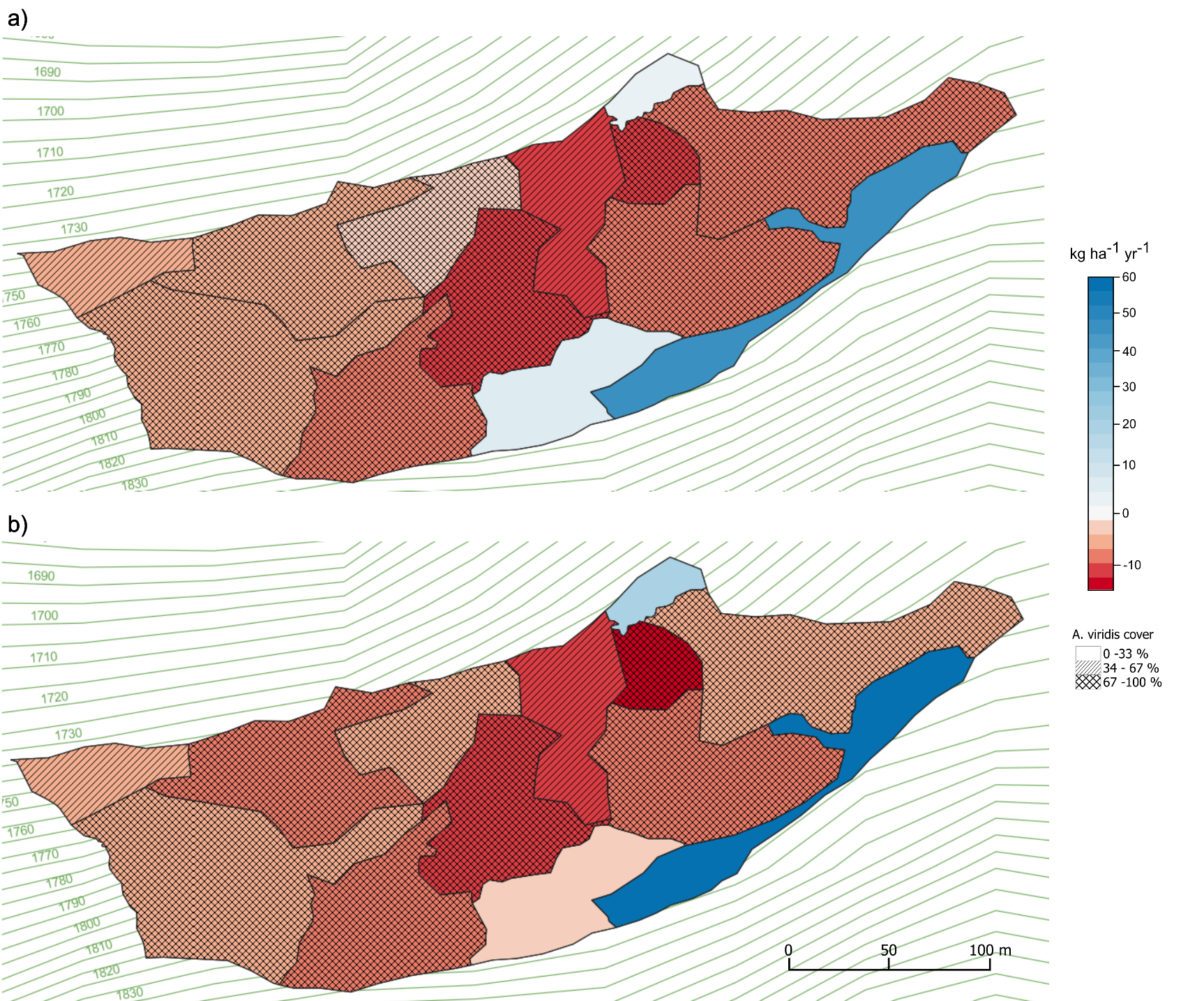
**Supplementary S8** –Nitrogen fluxes (50% quantile, kg ha^-1^ yr^-1^) of the vegetation patches of paddock 3in: a) 2019, and b) 2020.

**Supplementary S9** –Nitrogen fluxes (50% quantile, kg ha^-1^ yr^-1^) of the vegetation patches of paddock 4 in 2020.


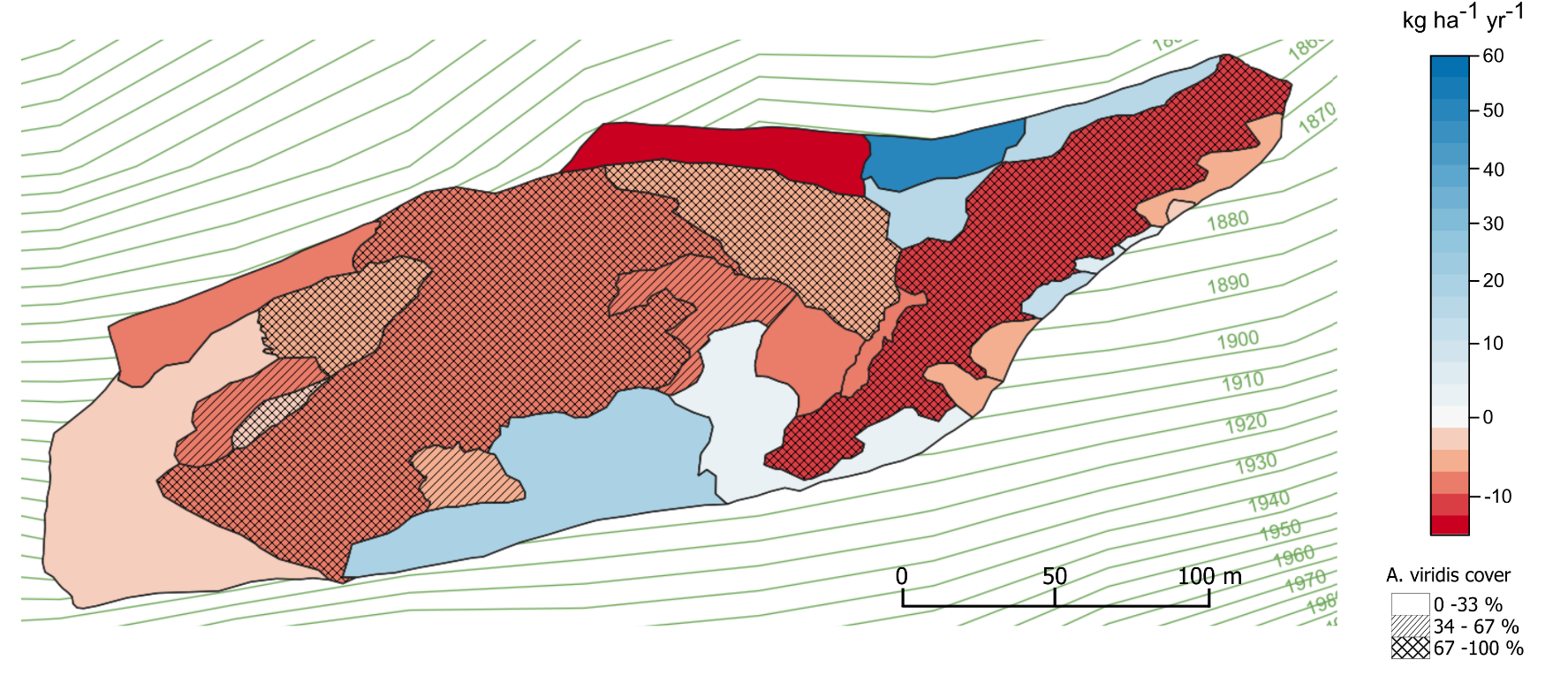

Supplement: Supplementary file 1 — Supplementary file1 (DOCX 17921 KB) [file 10705_2023_10282_MOESM1_ESM.docx]
